# Traumatic and Non-traumatic Radiculomedullary Compressions: A Comparative Analysis

**DOI:** 10.7759/cureus.71352

**Published:** 2024-10-13

**Authors:** Israël Maoneo, Antoine Beltchika, Teddy Ketani, Larrey Kasereka, Pierre Akilimali, Glennie Ntsambi

**Affiliations:** 1 Neurosurgery, University of Kisangani, Kisangani, COD; 2 Neurosurgery, University of Kinshasa, Kinshasa, COD; 3 Neurological Surgery, Makerere University, Kampala, UGA; 4 Nutrition, University of Kinshasa, Kinshasa, COD

**Keywords:** asia, non-traumatic spinal cord injury, radiculomedullary lesions, scim, spinal cord injuries, spinal surgery, traumatic spinal cord injury

## Abstract

Background

Radiculomedullary lesions represent a significant public health issue, with their epidemiological, clinical, and therapeutic characteristics varying depending on whether they are of traumatic or non-traumatic origin. The aim of this study was to compare clinical, therapeutic, and postoperative aspects between traumatic radiculomedullary lesions (TRML) and non-traumatic radiculomedullary lesions (NTRML).

Methods

This was a prospective cohort study conducted from 2020 to 2023 involving patients suffering from radiculomedullary lesions operated at the Department of Neurosurgery, University Teaching Hospital of Kinshasa. In addition to socio-demographic characteristics, the two patient groups - traumatic and non-traumatic - were compared based on clinical, therapeutic, and postoperative aspects using the American Spinal Injury Association (ASIA) and Spinal Cord Independence Measures (SCIM III) scores.

Results

We included 153 patients, with 73 traumatic cases (47.7%) and 80 non-traumatic cases (52.3%). TRMLs were predominantly caused by road traffic accidents (34%) and falls (11%), while NTRMLs were mainly due to disc herniations (22.2%) and tuberculosis (13.7%). The mean age for TRMLs was 35.4 ±12.8 years with a sex ratio of 3.5, compared to 50.7±15.9 years and a sex ratio of 1.1 for NTRMLs. TRMLs were more frequently located in the cervical region (32.8%) and the thoracolumbar junction (40%), whereas NTRMLs predominantly affected the thoracic (22.5%) and lumbar (63.7%) regions. Patients with NTRMLs had more incomplete lesions (98.7%) and better SCIM III scores at admission compared to TRMLs (p ˂ 0.001). TRMLs had more complete deficits 42 (57.3%) vs 1 (1.3%). Both groups significantly improved their ASIA and SCIM III scores postoperatively (p ˂ 0.001) but in a similar manner (Diff-in-diff: ASIA, p=0.955; SCIM, p=0.967). TRMLs developed more complications than NTRMLs (p˂0,001). Only five patients (11.6%) with ASIA A progressed to higher grades, and all remained dependent (SCIM III score ˂50). The average hospital stay was 89.2 ±74.2 days for TRMLs and 57.5±52.9 days for NTRMLs (p˂0.001).

Conclusion

This study revealed that TRMLs frequently affect young male individuals and are often located in the cervical region and thoracolumbar junction. In contrast, NTRMLs affect older individuals without gender preference and are usually found in the thoracic and lumbar regions. TRMLs often lead to complete deficits, pressure sores, urinary infections, and longer hospital stays compared to NTRMLs. Both patient groups showed significant postoperative improvement with no significant difference between them. However, patients with complete deficits showed less improvement in both groups.

## Introduction

Traumatic (TRML) and non-traumatic radiculomedullary lesions (NTRML) represent a significant public health concern due to their incidence and the devastating physical, psychological, and socio-economic impacts they cause [1]. These lesions are most commonly the result of compression rather than partial or complete transection, and may also arise from compromised blood flow, inflammatory processes, metabolic disorders, or exposure to toxins [2].

Globally, the incidence of radiculomedullary lesions, from all causes, is approximately 13 new cases per million people per year (ranging from 9 to 163.4 per million people per year) [1,3]. In the Democratic Republic of the Congo (DRC), the prevalence is around 216 (ranging from 181 to 296) per million people, with an incidence of 9 (ranging from 7 to 10) per 100,000 people [1]. The global prevalence of spinal cord lesions varies from 223 to 2525 per million people per year [1,4].

Traumatic or non-traumatic involvement of the spinal cord and nerve roots results in a disabling condition. This can lead not only to loss of sensation and motor function but also to dysfunction in multiple organs. The high treatment costs, prolonged recovery period, and loss of labour capacity have significant effects on both the individual and the family and also impose a substantial burden on society [3]. Mortality rates for radiculomedullary lesions vary between 4% and 18%, depending on the study series [5]. Overall, individuals with spinal cord injuries are two to five times more likely to die than healthy individuals [4].

There are several measures of postoperative functional outcomes that are integral to the comprehensive management of spinal cord injury patients. These include the American Spinal Injury Association (ASIA) scale [6], the Functional Independence Measure (FIM) score [7], and the Spinal Cord Independence Measures III (SCIM III) scores [8]. These and other measures help to assess the quality of care provided to spinal cord injury patients. The goal of treatment is not only the complete restoration of sensory-motor deficits but also the achievement of a certain level of independence in daily life activities [9].

Researchers agree that the epidemiological, clinical, and therapeutic characteristics of these lesions vary from region to region and depend on whether the origin is traumatic or non-traumatic [10]. TRMLs often affect young adult males, who are typically married, and frequently lead to complete neurological deficits. In contrast, NTRMLs tend to occur in older individuals, often females, and result in incomplete neurological deficits [11].

According to the World Health Organization (WHO), the world's population is ageing faster than in the past. By 2050, the number of people aged 60 and over in the world will have doubled to 2.1 billion people. The number of people aged 80 and over is expected to triple between 2020 and 2050 to reach 426 million [12]. This ageing of the world’s population will result in an increase in the number of cases of NTRML.

There is a debate on the postoperative outcome between TRMLs and NTRMLs among researchers. The majority of researchers maintain that there is no difference between traumatic and non-traumatic patients in terms of postoperative neurological and functional recovery [11, 13,14]. For other authors, post-therapeutic neurological and functional outcomes remain different between TRMLs and NTRMLs [15,16].

In addition, in the Democratic Republic of the Congo, people readily accept surgery to treat TRMLs. They claim that non-operative treatment leads to a good result in the case of NTRMLs. Faced with this divergence of view on post-therapeutic neurological and functional recovery among the authors, and considering the ageing of the world population as described by the WHO [12], which may lead to the worsening of cases of NTRML and the reluctance of the Congolese population with regard to surgical treatment in NTRML, we thought it was appropriate to undertake this study.

We aimed to compare the sociodemographic, clinical, and therapeutic aspects and postoperative functional recovery of patients with TRML AND NTRML using the ASIA and SCIM III scores as we wanted to know more about whether or not neurological and functional recovery depends on traumatic or non-traumatic origin.

## Materials and methods

Study design

This was an observational prospective cohort study conducted at the University Teaching Hospital of Kinshasa from January 1, 2020, to April 30, 2023. Data were prospectively collected during this period, encompassing all patients who underwent surgery for traumatic and non-traumatic radiculomedullary compressions.

Data Collection Procedure

We used the prospective collection documentary technique using hospitalisation and operating room registers as well as the data collection sheet.

Inclusion Criteria

All patients aged 10 years and above, admitted and operated on at the Vertebral-Medullary Injury Unit (VMIU) with a diagnosis of radiculomedullary compression syndrome (traumatic and non-traumatic) between January 1, 2020, and April 30, 2023, were included in the study.

Exclusion Criteria

Patients aged 10 years and above who were admitted and underwent surgery at VMIU were excluded from the study if their data could not be accessed or if they had previously undergone spinal surgery for unrelated reasons (for example, spinal fractures without radiculomedullary compression or spina bifida).

Scoring

In addition to various socio-demographic, clinical, and therapeutic variables (such as sex, age, admission date, date of surgery, date of discharge, occupation, presenting complaints, history, vital signs, cause of radiculomedullary compression, vertebral level of the lesion, neurological level at admission and discharge, treatment, complications, and discharge destination), we determined the ASIA and SCIM III scores of the patients preoperatively. The patients were then followed up postoperatively, with ASIA and SCIM III scores assessed monthly until discharge.

ASIA Score

The ASIA (American Spinal Injury Association) score is used to assess the severity of neurological injury. It comprises two components: motor and sensory. The motor score is out of 100, based on the assessment of five key muscle groups in the thoracic and pelvic limbs. Each muscle group is rated on a scale from 0 to 5:

0 = no movement; 1 = beginning of movement; 2 = movement without gravity; 3 = movement against gravity; 4 = movement against resistance; 5 = normal movement

The sensory score is out of 224, based on the assessment of 28 dermatomes. Each dermatome is rated as follows:

0 = no tactile or pain sensation; 1 = moderate sensation; 2 = normal sensation [6].

Based on these assessments, patients are classified into one of five grades: A, B, C, D and E. Grade A reflects a state of complete deficit, grades B, C, and D mean an incomplete neurological deficit and grade E is the normal state [17] (Table 1). 

**Table 1:** ASIA grades ASIA: American Spinal Injury Association

ASIA Grade	Type of Injury	Definition of type of Injury
Grade A	Complete	No motor or sensory function
Grade B	Incomplete	Sensory but no motor function is preserved below the level of injury
Grade C	Incomplete	Motor function is preserved, but majority of key muscles below the neurological level have a muscle grade <3
Grade D	Incomplete	Motor function is preserved, but the majority of key muscles below the neurological level have a muscle grade >3
Grade E	Normal	Motor and sensory functions are normal

SCIM III Score

The SCIM score evaluates recovery of independence following spinal cord injury. Three versions of this score exist (SCIM I, SCIM II, SCIM III), with SCIM III used in this study. It covers three functional domains:

Self-care (six items: feeding, upper body washing, lower body washing, upper body dressing, lower body dressing, personal hygiene), scored from 0 to 20; respiration and sphincter control (four items: respiration, bladder control, bowel control, and toilet use), scored from 0 to 40; and mobility, (1) In bed and the bathroom (three items: mobility in bed, transfers from bed to wheelchair or toilet, pressure sore prevention), scored from 0 to 10, and (2) indoor and outdoor mobility (six items: indoor movement, walking 10-100m, walking >100m, using stairs, transferring from wheelchair to car, and transfers from floor to wheelchair), scored from 0 to 30.

The total SCIM III score ranges from 0 to 100, with 0 indicating total dependence and 100 indicating full independence [18].

Outcomes

We evaluated two types of outcomes, primary and secondary. The primary outcome was assessed using the ASIA score for the neurological outcome and the SCIM III score for the functional result. Postoperative improvements in sensory and motor function were indicated by an upgrade in ASIA grade, while poor outcomes resulted in no change or a downgrade in ASIA grade. We noted whether there was postoperative neurological improvement between patients with traumatic radiculomedullary lesions (TRMLs) and those with non-traumatic radiculomedullary lesions (NTRMLs). Functional improvement was reflected by an increase of at least 10 points at discharge, indicating improved independence. Poor outcomes resulted in no change or a decrease in SCIM III score [19]. The secondary outcome included postoperative complications (including death and length of hospital stay) between the two groups.

Therapeutic Aspects

Surgical procedures: For TRMLs, decompression, and fixation techniques varied depending on the spinal region. Cervical segments were approached either anteriorly or posteriorly, depending on the case, and involved corpectomy and fixation with plates and screws or anterior odontoid screw fixation. Thoracic, thoracolumbar, and lumbar segments were approached posteriorly, with procedures such as simple decompression laminectomy or laminectomy with arthrodesis.

For NTRMLs, surgical techniques depended on the aetiology. Tumours were excised following laminectomy, with Saito's technique used for meningiomas [20]. In cases of instability, transpedicular fixation was performed. Treatment of Pott’s disease involved posterior decompression with laminectomy, interbody fusion, and transpedicular screw fixation. In cases with large paravertebral abscesses, drainage was performed to delineate lesion margins, followed by debridement and spinal realignment. Herniated discs were treated with discectomy following laminectomy, and in cases of spinal instability, fixation with transpedicular screws was performed.

Medical treatment: All patients received medical management, including analgesics, anti-inflammatory drugs, gastric acid inhibitors, postoperative corticosteroids, neurotropic drugs, anticoagulants, and antibiotics. For Pott's disease, patients received antitubercular treatment (isoniazid, rifampicin, ethambutol, and pyrazinamide) for 12 months, as recommended by the pulmonology team and guidelines.

Physiotherapy: All patients underwent physiotherapy to strengthen the paravertebral and limb muscles postoperatively until discharge, with some patients continuing rehabilitation at specialised centres.

Statistical analysis

Data were encoded using Microsoft Excel 2013 (Microsoft Corp., Redmond, WA). After ensuring the consistency of the database, the data were exported and analyzed using SPSS version 26 (IBM Corp., Armonk, NY) and STATA version 17 (StataCorp., College Station, TX). Descriptive statistics were presented in tables, with percentages for qualitative variables. Quantitative variables were expressed as mean ± standard deviation, assuming normal distribution.

We employed a fixed-effects linear regression panel model to analyze the evolution of recovery over time using ASIA and SCIM III scores. The difference-in-difference (Diff-in-Diff) approach was used to compare changes in ASIA and SCIM III scores between admission and discharge in both groups. The Diff-in-Diff coefficient indicated the average effect of the cause on the treated individuals after adjusting for age and sex. The dependent variable was considered as the improvement in ASIA score (transition from a lower stage to a higher stage) and the acquisition of some autonomy following a 10-point increase in SCIM III score at discharge.

A p-value of less than 0.05 was considered to be a threshold of statistical significance.

## Results

Causes of spinal cord injury

A total of 153 patients underwent surgery for radiculomedullary compression, with 80 (52.3%) being non-traumatic and 73 (47.7%) being traumatic. The traumatic injuries were mainly due to road traffic accidents (34%) and falls (11%). Non-traumatic lesions were primarily caused by disc herniation (22.2%), tuberculosis (13.7%), tumours (9.8%), and spinal canal stenosis (6.6%) (Table 2).

**Table 2:** Causes of spinal cord injury Data presented as n (frequency), % (percentage).

Causes	n	%
Traumatic	73	47.7
Road accidents	52	34
Falls	17	11
Firearms and/or knives	4	2.7
Nontraumatic	80	52.3
Herniated discs	34	22.2
Tuberculous spondylodiscitis	21	13.7
Tumors	15	9.8
Canal stenosis	10	6.6

Socio-demographic, clinical, and therapeutic characteristics

Patients with TRML were younger than those with NTRML. The mean age was 35.4 ± 12.8 years for traumatic cases and 50.7 ± 15.9 years for non-traumatic cases (p<0.001). Males were more affected than females in both groups, with sex ratios of 3.5 and 1.1, respectively. However, the number of females with NTRML was close to that of males (42 vs 38). Most traumatic lesions were located in the cervical (32.8%) and thoracolumbar (40%) regions (p<0.001). In contrast, non-traumatic lesions were predominantly thoracic (22.5%) and lumbar (63.7%) (p<0.001). Patients with NTRML had better average ASIA and SCIM III scores at admission compared to those with TRML (p<0.001). Many patients in the traumatic group had a complete deficit (ASIA score A) compared to the non-traumatic group (p<0.001) and; they were mostly dependent (SCIM III score ≤40, p <0.001). Spinal fixation and corpectomy were more commonly performed in traumatic cases, while non-traumatic cases more frequently underwent discectomies. Patients with Pott’s disease underwent posterior decompression via laminectomy, debridement, posterior abscess drainage, and interbody fusion with transpedicular screw fixation, connected by rods. All patients received medical therapy and physiotherapy. Tuberculosis cases were treated with a 12-month course of antitubercular medication (Table 3).

**Table 3:** Socio-demographic, clinical and therapeutic characteristics Data presented as n (frequency), mean, SD (standard deviation), p-value by t-test, and chi-square test. *These surgical procedures are only performed for nontraumatic radiculomedullary lesions and could not be used to compare the two patient groups. ASIA: American Spinal Injury Association; SCIM III: Spinal Cord Independence Measures III

Characteristics	TRML	NTRML	p-value
	n	n	
Age (Mean in years) (SD)	35.4(12.8)	50.7(15.9)	<0.001
Sex			
Male	57	42	<0.001
Female	16	38	<0.001
Sex ratio	3.5	1.1	
Segments			
Cervical	24	8	<0.001
Thoracic	12	18	0.346
Thoracolumbar	30	3	<0.001
Lumbar	7	51	<0.001
ASIA score			
A	42	1	<0.001
B	19	19	0.745
C	15	6	0.889
D	6	24	<0.001
E	0	21	<0.001
Mean ASIA (SD)	1.6 (0.9)	3.5(1.2)	<0.001
SCIM III score			
≤10	14	1	<0.001
11 - 40	47	19	<0.001
41 - 70	4	17	0.007
71 - 100	4	43	<0.001
Mean SCIM III (SD)	31.9 (19.9)	71.2(26.8)	<0.001
Surgical procedures			
Laminectomy	10	21	0.54
Laminectomy + fixation	45	28	<0.001
Laminectomy + discectomy	0	18	<0.001
Corpectomy + fixation	18	4	<0.001
Laminectomy + mass excision*	0	8	-
Laminectomy + mass excision + fixation*	0	1	-

Evolution of ASIA score over time in two groups

Tables 4-5 indicate that ASIA scores improved in both groups from admission to discharge. The interaction between cause and time (Cause #Time) is significant (p<0.001). The main effect of the model is significant for both the time and cause of radiculomedullary lesions (p<0.001). Patients with ASIA A score at admission showed less improvement in neurological function in both groups, with no significant difference (p=0.714). Patients with ASIA B scores partially improved (20 improved vs 18 remained stationary) similarly in both groups. Patients with ASIA C and ASIA D significantly improved in a similar manner (p=0.517) (Table 6). The "Diff-in-Diff" coefficient indicates the absence of an effect of cause on the operated patients (p=0.995) (Table 7).

**Table 4:** Evolution of the average ASIA scores from admission to discharge Data presented as n (frequency), mean, SD (standard deviation) TRML: traumatic radiculomedullary lesions; NTRML: non-traumatic radiculomedullary lesions

Patient Groups		Admission	Discharge
NTRML	n	80	80
	Mean	3.56	4.15
	SD	1.16	1.20
TRML	n	73	73
	Mean	1.67	2.26
	SD	0.94	1.40
Total	n	153	153
	Mean	2.66	3.25
	SD	0.87	0.92

**Table 5:** Evolution of different ASIA scores from admission to discharge Data presented as n (frequency), % (percentage) ASIA: American Spinal Injury Association; TRML: traumatic radiculomedullary lesion; NTRML: non-traumatic radiculomedullary lesion

Patient Groups	ASIA									
	Admission; n (%)					Discharge; n (%)				
	A	B	C	D	E	A	B	C	D	E
TRML	42 (58)	19 (26)	6 (8)	6 (8)	0 (0)	37 (51)	10 (14)	8 (11)	11 (15)	7 (9)
NTRML	1 (1)	19 (24)	15 (19)	24 (30)	21 (26)	1 (1)	13 1(6)	8 (10)	13 (16)	45 (56)
Total	43 (28)	38 (25)	21 (14)	30 (19)	21 (14)	38 (25)	23 (15)	16 (10)	24 (16)	52 (34)

**Table 6:** Comparison of neurological impairment (ASIA A, B, C, D) at discharge Data presented as n (frequency); chi-square test is used to calculate the p-values ASIA: American Spinal Injury Association; TRML: traumatic radiculomedullary lesion; NTRML: non-traumatic radiculomedullary lesion

ASIA	Neurological impairment at discharge	Patient Groups		Total	p-value
		NTRML (n)	TRML (n)		
A	Improved	0	5	5	0.714
	Unimproved	1	37	38	
	Sub-total	1	42	43	
B	Improved	10	10	20	1.000
	Unimproved	9	9	18	
	Sub-total	19	19	38	
C	Improved	11	4	15	0.780
	Unimproved	4	2	6	
	Sub-total	15	6	21	
D	Improved	19	4	23	0.517
	Unimproved	5	2	7	
	Sub-total	24	6	30	
Total		59	73	132	

**Table 7:** Comparison of mean ASIA scores before and after surgery R-square: 0.38; inference:  p<0.05 Means and standard errors are estimated by linear regression. ASIA: American Spinal Injury Association; TRML: traumatic radiculomedullary lesions; NTRML: non-traumatic radiculomedullary lesions;

Dependent Variable	ASIA	Standard Error	t-test	p-value
Before surgery				
TRML	1.671			
NTRML	3.562			
Diff (N-T)	1.891	0.192	9.84	0.000
After surgery				
TRML	2.260			
NTRML	4.150			
Diff (N-T)	1.890	0.192	9.83	0.000
Diff-in-Diff	-0.002	0.272	0.01	0.955

Evolution of SCIM III score over time in two groups

Table 8 shows that patients' SCIM III scores improved over time from admission to discharge. The interaction between cause and time (Cause #Time) is significant (p<0.001). The main effect of the model is significant for time and for the cause of the radiculo-medullary lesion (p<0.001). Patients with complete impairment had less progress in both groups and remained dependent at the end of hospitalization (SCIM III score ˂50) (Table 9). The "Diff-in-Diff" coefficient indicates the absence of an effect of the cause on the operated individuals (p=0.967) (Table 10).

**Table 8:** Evolution of the average SCIM III scores from admission to discharge SCIM III: Spinal Cord Independence Measures III; TRML: traumatic radiculomedullary lesion; NTRML: non-traumatic radiculomedullary lesion Data presented as n (frequency), mean, SD (standard deviation)

Patient Groups		Admission	Discharge
NTRML	n	80	80
	Mean	71.18	82.26
	SD	26.83	26.99
TRML	n	73	73
	Mean	31.82	43.38
	SD	19.97	29.04
Total	n	153	153
	Mean	52.46	63.71
	SD	30.82	34.03

**Table 9:** Comparison of SCIM III scores of patients with complete impairment at discharge Data presented as n (frequency); chi-square test was used to calculate p-value. SCIM III: Spinal Cord Independence Measures III; TRML: traumatic radiculomedullary lesion; NTRML: non-traumatic radiculomedullary lesion

Patient groups	Complete impairment	SCIM III						Total	p-value
		Admission		Discharge					
				improved		Unimproved			
		n	(SCIM)	n	(SCIM)	n	(SCIM)		
TRML	Complete tetraplegia	10	6/100	0	-	10	6/100	10	-
						27	32/100	32	
TRML	Complete paraplegia	32	32/100	4	33/100	27	32/100	32	0.714
				1	60/100				
NTRML	Complete paraplegia	1	32/100	0	-	1	32/100	1	
Totals		43		5		38		43	

**Table 10:** Comparison of mean SCIM III scores before and after surgery R-square: 0.38; inference:  p<0.05 Means and standard errors are estimated by linear regression. SCIM III: Spinal Cord Independence Measures III; TRML: traumatic radiculomedullary lesion; NTRML: non-traumatic radiculomedullary lesion

Dependent Variable	SCIM	Standard Error	t-test	p-value
Before				
TRML	32.67			
NTRML	71.00			
Diff (N-T)	38.32	4.206	9.11	0.000
After				
TRML	44.14			
NTRML	82.72			
Diff (N-T)	38.57	4.206	9.17	0.000
Diff-in-Diff	0.249	5.948	0.04	0.967

Hospitalization and discharge characteristics

Pressure ulcers and urinary tract infections were more frequently observed in traumatic patients than in non-traumatic patients (p<0.001 and p<0.001, respectively). Surgical site infection was equally distributed in both groups. Hospital stay was longer in the traumatic group than in the non-traumatic group (p=0.001) (Table 11).

**Table 11:** Hospitalization and discharge characteristics Data presented as n (frequency), means and SD (standard deviation). The strength of association between variables was calculated by OR (odds ratio) and CI (confidence interval). *OR (CI): 14.7 (3.3-85.6), ** OR(CI): 4.8 (2.4-9.6) TRML: traumatic radiculomedullary lesion; NTRML: non-traumatic radiculomedullary lesion

Characteristics	TRML	NTRML	p-value
	n	n	
Complications			
Pressure ulcers	20	2	<0.001*
Surgical site infections	11	17	0.323
Urinary tract infections	45	20	<0.001**
Death	5	5	0.881
Hospital stay in days; mean(SD)	89.2(74.2)	57.5(52.9)	0.001

## Discussion

Socio-demographic and clinical characteristics at admission

Traumatic injuries were predominantly associated with road traffic accidents (34%) and falls (11%). Non-traumatic injuries were primarily due to degenerative conditions, such as herniated discs (22.2%). The mean age of those with traumatic injuries was 35.4±12.8 years, with a male-to-female ratio of 3.5. In our series, the mean age of patients with non-traumatic injuries was 50.7±15.9 years, with a male-to-female ratio of 1.1. Most traumatic lesions were located in the cervical region (32.8%) and at the thoracolumbar junction (40%) (p < 0.001), while non-traumatic injuries were predominantly found in the lumbar (63.7%) and thoracic (22.5%) regions. The majority of patients in the trauma group had a complete deficit (57.5%), while only one non-traumatic patient had an ASIA A score.

These findings are consistent with data from previous studies. TRMLs are more common among younger male individuals [15]. In their respective studies, Null et al. [21], Ones et al. [22], and Gaddour et al. [15] reported mean ages of 41.8 ±15.5 years, 35.8+15 years, and 34 years, with male-to-female ratios of 5.4:1, 4.4:1, and 2.4:1, respectively.

NTRMLs typically affect older adults in their fifties, with both sexes being equally affected. In studies focusing on non-traumatic injuries [23], Gaddour et al. [15], Gedde et al. [14], and Ekouele et al. [24] documented mean ages of 48.5 years, 52 years, and 49.2±17.3 years, with male-to-female ratios of 0.8:1, 1.3:1, and 1.2:1, respectively.

Overall, spinal cord injuries are located in the cervical region in 45.3% of cases, thoracic in 39.9%, and lumbosacral in 14.8% [25]. Traumatic spinal cord injuries are often found in the cervical region [13], while non-traumatic injuries are typically located in the thoracic segment (71.3%) [26]. However, thoracic involvement is often associated with age, being more common in patients aged 31 to 45 years, while cervical involvement shows two peaks: one between 15 and 30 years and another in those over 60 years. For spinal cord compressions of tumour origin, the thoracic spine is the most common site, whereas lumbar or cervical locations predominate in degenerative conditions [21].

In a study conducted by Pefile et al. in South Africa, non-traumatic injuries affected the thoracic (53%) and lumbar (39%) regions of the spine, while traumatic injuries mainly involved the thoracic (46%) and cervical (34%) segments [27]. In contrast, non-traumatic injuries were located in the thoracic (16%) and lumbar (54.7%) regions in the Gbadamosi et al. study from Ghana [28].

Neurological impairment is often incomplete in non-traumatic lesions but frequently complete in traumatic radiculomedullary lesions [23]. In the study by Gedde et al. [14], 36 traumatic patients (35%) had an ASIA A classification compared to seven non-traumatic cases (10%) (p=0.001). Alito et al. recorded six traumatic cases (20%) with ASIA A compared to two non-traumatic patients (2%) [13].

The degree of neurological impairment in non-traumatic injuries is primarily determined by the underlying cause. It is severe in cases of infectious and tumour origin but less severe in degenerative conditions. A study by New reported a high ASIA score for degenerative pathologies (D-E: 65%) and a low ASIA score for infections (A-C: 62.1%) [29]. The high ASIA score recorded in our cohort may be attributed to the predominance of degenerative lesions.

Postoperative course

In this study, both ASIA and SCIM III scores improved similarly in both groups from admission to discharge. The difference-in-difference coefficients for these two scores did not reveal any significant differences (p=0.955 and p=0.967, respectively). In the traumatic group, we observed a higher incidence of pressure sores, urinary tract infections, and prolonged hospitalisation.

Radiculomedullary lesions, whether traumatic or non-traumatic, generally show favourable postoperative outcomes. Some authors have found no significant difference in functional recovery between the two groups at the end of treatment [11,14]. Gedde et al. [14] documented that 26% of patients with traumatic lesions and 18% of non-traumatic cases improved their neurological function (p=0.438).

Patients with an ASIA A score on admission, whether traumatic or non-traumatic, demonstrated less neurological improvement and remained more dependent compared to those with ASIA B, C, and D scores. This finding is consistent with previous studies [11,22]. Of the 43 patients with ASIA A documented in the series by Gedde et al. [14], only four (9.3%) showed significant improvement (3/36 traumatic, 1/7 non-traumatic), while 39 maintained their initial neurological status. Indeed, an ASIA grade A reflects complete impairment due to a severe radiculomedullary lesion, which can adversely affect or delay postoperative functional recovery. The cause of the radiculomedullary lesion appears to be less critical to the improvement of the patient's neurological function compared to the level and severity of the initial spinal cord injuries [14,16].

In previous studies, it has been reported that pressure ulcers, urinary tract infections, and prolonged hospitalization predominately in traumatic patients [25,30]. We believe that the predominance of complete lesions in traumatic patients, which necessitates prolonged bed rest, may account for these complications and extended hospital stays.

Limitations

The small sample size does not allow us to generalise our conclusions. Additionally, the study was not conducted in the general population of the city of Kinshasa, it was based only on data from one facility, Kinshasa Teaching Hospital, this does not allow to generalise the findings. Finally, the study was conducted in a specific geographical region, the city of Kinshasa, which may limit the generalizability of the results to other populations or regions of the Democratic Republic of the Congo. We do not generalise the conclusions to other countries of the world with different demographic characteristics.

## Conclusions

This study revealed that TRMLs predominantly affect young male individuals and are mainly located in the cervical region and the thoracolumbar junction. In contrast, NTRMLs primarily affect older adults with no gender preference and are usually found in the thoracic and lumbar segments. TRMLs often result in complete deficits, pressure ulcers, urinary tract infections, and longer hospital stays compared to NTRMLs. Overall, both types of lesions tend to improve over time post-operatively without any significant difference. However, patients with ASIA A scores, regardless of whether the injury is traumatic or non-traumatic, have difficulty improving their initial neurological status and tend to remain dependent.
